# Relationship between Determinants of Food Choices and Socioeconomic and Demographic Factors of Individuals with Hepatitis B and C in the Amazon Region

**DOI:** 10.3390/foods12122359

**Published:** 2023-06-13

**Authors:** Manuela Maria de Lima Carvalhal, Rayzza Marcelly Jesus da Silva, Tayna Carvalho Pereira, Camila Rodrigues Monteiro, Daniela Lopes Gomes, Juarez Antônio Simões Quaresma

**Affiliations:** 1 Nucleus of Tropical Medicine, Federal University of Pará, Belém 66055-240, PA, Brazil; 2 Institute of Health Sciences, Federal University of Pará, Belém 66075-110, PA, Brazil; raymsilva14@gmail.com; 3 Postgraduate Program in Biology of Infectious and Parasitic Agents, Federal University of Pará, Belém 66075-110, PA, Brazil; taynacarvalhop@outlook.com; 4 Hospital North Coast Region, São Paulo 11672-032, SP, Brazil; camilaflantis@hotmail.com; 5 Postgraduate Program in Neuroscience and Behavior, Behavior Theory and Research Nucleus, Federal University of Pará, Belém 66075-110, PA, Brazil; danielagomes@ufpa.br; 6 Department of Pathology, State University of Pará, Belém 66087-662, PA, Brazil; 7 School of Medicine, São Paulo University, São Paulo 01246-903, SP, Brazil

**Keywords:** hepatitis C, hepatitis B, feeding behavior

## Abstract

Knowing the determinants of food choices allows the nutritionist to develop more assertive guidelines considering biopsychosocial factors to produce effective changes in eating practices. This cross-sectional, descriptive, and analytical study aimed to test the correlation between the determinants of food choices and the socioeconomic and demographic factors of individuals with hepatitis B and C. Patients with hepatitis B and/or C aged between 20 and 74 years were evaluated from August 2020 to August 2021. Their socioeconomic and demographic data and clinical data were collected, and The Eating Motivation Survey (TEMS) was applied. A total of 145 individuals were evaluated, with a mean age of 53.54 ± 12.14 years. There were positive weak correlations between gender (*p*^2^ = 0.193; *p* = 0.020) and age (*p*^2^ = 0.177; *p* = 0.033) with the scale “preference”; negative correlations between age and the scales “price” (*p*^2^ = −0.204; *p* = 0.014) and “emotion control” (*p*^2^ = −0.168; *p* = 0.044); negative correlations between education and the scales “convenience” (*p*^2^ = −0.172; *p* = 0.039) and “social norms” (*p*^2^ = −0.206; *p* = 0.013); and income showed a negative correlation with “price” (*p*^2^ = −0.208; *p* = 0.012) and a positive correlation with “weight control” (*p*^2^ = 0.186; *p* = 0.025). These findings contribute to the development of more realistic and feasible eating strategies that favor food autonomy.

## 1. Introduction

Hepatitis B and C are viral infections that constitute obstacles to the maintenance of public health worldwide due to the severity of the liver damage caused [1]. In Brazil, the Notifiable Diseases Information System (*Sinan*) [2] recorded 718.651 confirmed cases of viral hepatitis throughout the national territory between 2000 and 2021, 36.8% of which were infections by the hepatitis B virus (HBV) and 38.9% by the hepatitis C virus (HCV).

Due to the metabolic alterations observed in patients with hepatitis, it is important to implement measures that promote changes in lifestyle, which mainly include adequate nutrition and physical exercise [3]. According to European Association for the Study of the Liver (EASL) [4] and Jalolov, Imamova and Kobilzhonova [5], generally, a healthy diet with a variety of foods, balanced in terms of proteins, fats and carbohydrates is advisable for all individuals with liver disease, with adequate food consumption being more important than dietary restrictions.

In this context, in people with hepatitis C, Batool and Kausar [6] showed that health-related behaviors improved significantly after the diagnosis of the disease, and that there was a significant association between good adherence to clinical treatment, diet and eating behavior. 

Although there are studies that have assessed the quantitative food consumption of people with viral hepatitis [7,8,9,10], there is no research that investigated the factors that influence the eating behavior of these individuals. According to Leng et al. [11], among the aspects that interfere with eating behavior, we can mention genetic, epigenetic and metabolic factors, in addition to conditions related to the environment such as sociocultural elements (family and social influence, purchasing power), cognitive-affective (stress perception, health attitude, anxiety and depression) and availability of dietary components (highly palatable foods, for example). Van Lenthe, Jansen and Kamphuis [12] mentioned that socioeconomic factors play a key role in food choices, since they directly influence the availability and acquisition of food.

Therefore, the importance of knowing the determinants of food choices is emphasized, since, according to Moraes and Alvarenga [13] and Rempe et al. [14], it allows the nutritionist to elaborate more assertive guidelines considering biopsychosocial factors, in order to provide strategies for effective changes in eating practices.

In this context, The Eating Motivation Survey (TEMS) developed by Renner et al. [15] contemplates various contexts relevant to food choices by observing 15 basic dimensions that influence the act of eating. To date, no studies have applied TEMS in individuals with viral hepatitis; however, Moraes et al. [16] found that the dimensions health, habits, natural concerns, and need and hunger had a considerable influence on the food choices of adults in two Brazilian cities with socioeconomic differences.

In the study by Pechey et al. [17], the authors aimed to assess whether differences in motivations for certain foods can contribute to socioeconomic differences in consumption in the general population, and observed that participants in the higher income group consider the health and weight control dimensions of TEMS as more frequent motivating factors, and price and convenience as less frequent factor, when compared to lower income participants.

Konttinen et al. [18] investigated how sociodemographic characteristics are associated with eating motives using the Food Choice Questionnaire and observed that younger groups make choices based on short-term motivations (mood control, convenience, price and palatability), while older individuals take long-term motivations into account (health, weight control and ethical concerns). Thus, sociodemographic characteristics have a strong influence on food choices. 

In this context, the scarcity of studies on the identification of food choices’ determinants of people with viral hepatitis is highlighted, in addition to the relationship with the socioeconomic factors of these individuals. Therefore, as we are aware of the importance, it is expected that the present study will make it possible to clarify the food choice motivations of individuals with viral hepatitis, raising the hypothesis that socioeconomic and demographic differences are reflected in the determinants of food choices. In this sense, it is expected that the level of education is correlated with scales related to the practicality of food, income is correlated with the price scale and age is correlated with the preference and price scales. Thus, the present study aimed to assess the correlation between the determinants of food choices and the socioeconomic and demographic factors of individuals with hepatitis B and C.

## 2. Materials and Methods

### 2.1. Study Type and Localization

This is a cross-sectional, descriptive, and analytical study, carried out from August 2020 to August 2021, at the Clinical Specialties Outpatient Clinic in a Reference Center in Belém, Pará.

### 2.2. Sample and Inclusion and Exclusion Criteria

Non-probabilistic convenience sampling was performed with patients diagnosed with viral hepatitis B and/or C.

The participants included in the research were between 20 and 74 years old (age range from adults to the elderly, according to the World Health Organization), diagnosed with viral hepatitis B and/or C who agreed to sign the Informed Consent Form (ICF) for data collection.

Exclusion criteria included people with liver disease caused by viral agents other than virus B and/or C; pregnant women and nursing mothers; patients with food allergies and intolerances; chronic renal failure patients; patients with neoplasms; edematous patients (lower and upper limbs and ascites); clinical intercurrence that made it impossible to apply the research form; and withdrawal of participation, even after signing the ICF.

### 2.3. Data Collection

#### 2.3.1. Socioeconomic and Demographic Data and Clinical Characterization

A form containing questions on socioeconomic and demographic characteristics was applied through interviews, addressing the questions of: age, gender, the patient’s origin (Capital/Metropolitan Region, another municipality in Pará or outside the State), level of education (with or without higher education) and family income in minimum wages (considering the current value of the year of 2020, equal to BRL 1.045).

Regarding clinical characteristics, the type of hepatitis (B and/or C) was verified from the patient’s chart.

#### 2.3.2. The Eating Motivation Survey

To assess food choices, the TEMS created by Renner et al. [15], translated and validated into Brazilian Portuguese by Moraes and Alvarenga [13], was applied. This instrument consists of a Likert-type scale with alternatives whose score ranges from 1 (never) to 5 (always), which started with the statement “I eat what I eat…” for all items.

The survey is composed, in its reduced version, of 45 items, distributed in 15 scales related to eating motivations. All scales contain 3 items each, which when added together, result in a score that ranges from 1 to 15 points:

(1) Preference: individual eating desires;
(2) Habits: familiarity with a certain food;
(3) Need and Hunger: the satisfaction that food can provide;
(4) Health;
(5) Convenience: the practicality that the consumption of a certain food can have;
(6) Pleasure;
(7) Traditional Food: for example, family traditions regarding certain foods;
(8) Natural Issues: such as the preference for organic products;
(9) Socialization;
(10) Price;
(11) Visual Attraction;
(12) Weight Control;
(13) Emotional control;
(14) Social Norms;
(15) Social Image.

### 2.4. Data Analysis 

The collected data are presented using measures of central tendency and variation. The G or Chi-Square independence test was used, followed by residual analysis, to test the association between the different categories of a variable in independent groups. Spearman’s correlation test was used to test correlations between TEMS scales and socioeconomic and demographic factors.

Variables that showed a statistically significant correlation in the bivariate analyses (*p* < 0.05) were included in the linear regression model. In the first regression analysis, the scale “price” was considered as the dependent variable and age (years), gender (male or female), income (up to one; from two to three; from four to five; above five), education (with or without higher education) and origin (capital or metropolitan region; another municipality in Pará; outside the state of Pará) as co-variables.

For the second regression analysis, the “emotion” scale was considered as the dependent variable and the co-variables were age (years); gender (male or female); income, considering the current value of the year of 2020, equal to BRL 1.045 (up to one; from two to three; from four to five; above five); education (with or without higher education); and origin (capital or metropolitan region; another municipality in Pará; outside the state of Pará).

In the third regression analysis, the dependent variable was the “social norms” scale and the co-variables were education (with or without higher education), gender (male or female), age (years), income (up to one; from two to three; from four to five; above five) and origin (capital or metropolitan region; another municipality in Pará; outside the state of Pará).

All tests were performed using the Statistical Package for Social Sciences software, version 21, using a statistical significance level of *p* < 0.05.

### 2.5. Ethical Aspects

The current research is part of the project entitled “Type 2 diabetes mellitus, cardiovascular risk and factors associated with metabolic syndrome in patients diagnosed with viral hepatitis treated at a reference center in the Amazon”, approved by the Ethics Committee under approval numbers 4.120.268 (29 June 2020) and 4.946.840 (1 September 2021) (for extension of data collection), complying with the legal requirements of Resolutions 466/12 and 510/16 of the Brazilian National Health Council and the Declaration of Helsinki.

## 3. Results

The study included 145 individuals, with a mean age of 53.54 ± 12.14 years. It can be observed from Table 1 that the majority of participants were female (53.10%; *p* = 0.455), were aged 45 to 59 years and 60 to 74 years (37.93%), came from the capital or a metropolitan region (78.62%), did not attain a higher education (82.07%), had a family income below the minimum wage (58.62%) and were diagnosed with hepatitis C virus infection (71.03%).

As for the TEMS scales (Table 2), the determinants with the highest mean score for the food choices of individuals with viral hepatitis were “habits” (12.43 ± 2.24), followed by the scales “preference” (12.11 ± 2.60), “need and hunger” (11.19 ± 2.42), “health” (10.66 ± 2.60) and “weight control” (8.19 ± 3.24) according to the five aspects with the highest relevance in food choices. On the other hand, it was found that the lowest mean score determinants were “social norms” (6.23 ± 2.42), “socialization” (5.99 ± 2.72), “visual attraction” (4.23 ± 2.20), “emotional control” (4.19 ± 2.27) and “social image” (3.60 ± 1.23). 

Table 3 presents the correlations between the TEMS scales and socioeconomic and demographic factors. Positive correlations were observed between gender (r^2^ = 0.193; *p* = 0.020) and age (r^2^ = 0.177; *p* = 0.033) with the “preference” scale. In addition, there were negative correlations between age and the scales “price” (r^2^ = −0.204; *p* = 0.014) and “emotional control” (r^2^ = −0.168; *p* = 0.044); education showed negative correlations with the scales “convenience” (r^2^ = −0.172; *p* = 0.039) and “social norms” (r^2^ = −0.206; *p* = 0.013); and income showed a negative correlation with “price” (r^2^ = −0.208; *p*= 0.012) and a positive correlation with “weight control” (r^2^ = 0.186; *p* = 0.025).

The variables that showed statistically significant weak correlations in the bivariate analyses were included in the multiple linear regression model. In Table 4, it can be observed that the correlation between the “price” scale and age remained (β = −0.199; R^2^ = 0.033; CI −0.102; −0.010; *p* = 0.017), regardless of gender (β = −0.190; R^2^ = 0.029; CI −0.100; −0.007; *p* = 0.023), income (β = −0.180; R^2^ = 0.064; CI −0.097; −0.005; *p* = 0.029), education (β = −0.188; R^2^ = 0.061; CI −0.099; −0.007; *p* = 0.024) and origin (β = −0.189; R^2^ = 0.054; CI −0.102; −0.005; *p* = 0.031).

When evaluating Table 5, it can be observed that the correlation between the scale “emotional control” and age remained significant (β = −0.234; R^2^ = 0.048; CI −0.074; −0.014; *p* = 0.005), regardless of gender (β = −0.220; R^2^ = 0.052; CI −0.071; −0.011; *p* = 0.008), income (β = −0.217; R^2^ = 0.049; CI −0.071; −0.010; *p* = 0.009), education (β = −0.215; R^2^ = 0.042; CI −0.071; −0.009; *p* = 0.011) and origin (β = −0.207; R^2^ = 0.036; CI −0.071; −0.006; *p* = 0.020).

In Table 6, it can be observed that the correlation between the “social norms” scale and education remained (β = −0.201; R^2^ = 0.034; CI −2.278; −0.245; *p* = 0.015), regardless of gender (β = −0.201; R^2^ = 0.027; CI −2.282; −0.241; *p* = 0.016), age (β = −0.216; R^2^ = 0.056; CI −2.367; −0.350; *p* = 0.009), income (β = −0.215; R^2^ = 0.050; CI −2.574; −0.128; *p* = 0.031) and origin (β = −0.216; R^2^ = 0.046; CI −2.585; −0.134; *p* = 0.030).

## 4. Discussion

The present study assessed the relationship between determinants of food choices and socioeconomic and demographic characteristics of individuals with hepatitis B and C.

Regarding the characterization of the sample, it was observed that most participants were female, in contrast to the data in the *Boletim Epidemiológico de Hepatites Virais* [Epidemiological Bulletin of Viral Hepatitis] [2] in which men represented the majority of individuals with infection by both viral agents. On the other hand, a study carried out in all Brazilian regions showed that women represent the majority of users of health services and that men show greater resistance, with the demand appearing only when they are older or in the presence of symptoms [19]. Therefore, this suggests that, in the present study, females may have been more concerned about health, contributing to a higher percentage of diagnosis and monitoring of liver diseases.

It was also observed that most Individuals were aged between 45 and 59 years and 60 and 74 years and were from the capital or metropolitan region. The data from the Epidemiological Bulletin of Viral Hepatitis [2] indicate that the predominant age group for hepatitis B is between 45 and 54 years, and for hepatitis C it is individuals over 60 years. In addition, the Bulletin also mentions that most cases of HCV infections occur in the state capitals [2], corroborating the findings of the current study.

In addition, the participants predominantly did not have higher education and had a family income below the minimum wage. De Farias et al. [20] highlighted that education and income are related to the understanding of health problems, confirming that underprivileged individuals in these aspects are more susceptible to viral infections. In addition, most participants were diagnosed with hepatitis C virus infection. According to the Epidemiological Bulletin of Viral Hepatitis [2], most cases of viral hepatitis reported in the period from 2000 to 2021 were hepatitis C, corroborating the present study. 

Regarding the mean TEMS scores, it was observed that the highest mean score for the food choices of individuals with viral hepatitis were “habits”, “preference” and “need and hunger”. There are no previous studies that evaluated the determinants of food choices of individuals with viral hepatitis; however, we hypothesized that the participants place a greater importance on commonly performed eating practices (habits and preferences), due to concern about consuming foods that can aggravate their liver disease, and on cultural habits and psychological factors. In addition, the high score for “need and hunger” demonstrates the importance of considering the socioeconomic aspects involved with food.

Based on the weak correlation analysis between socioeconomic and demographic factors and the TEMS scales, a direct correlation was found between gender and the preference scale, demonstrating that this scale is more relevant for women than for men in terms of food choices. There are no previous studies that have evaluated these relationships in people with viral hepatitis; however, Renner et al. [15], when developing the TEMS, observed that women are more influenced by food choices than men, as they have higher scores in 10 of the 15 determinants, among which is preference.

A direct correlation was also observed between age and the preference scale in food choices. On the other hand, there was an inverse correlation between age and the price and emotional control scales. From the linear regression analyses, the inverse correlations remained, regardless of gender, income, education and origin.

These findings are similar to those from the study by Rempe et al. [14] in an elderly population, which verified that preference was the main determinant for food choices; in addition, price and emotional control were among the last five scales to be considered. Renner et al. [15] found that younger people, when compared to people of advanced age, have their eating behavior guided by short-term motives, which are reflected in the scales of emotional control and price, as these are more immediate issues that need quick resolutions.

Thus, the importance of individual tastes for the food choices of the elderly is understood, considering that this stage of life is usually marked by illnesses, reducing the functional and cognitive capacity of these individuals, allowing them to enjoy food as a moment of pleasure and comfort, which may boost the maintenance of healthy eating habits [21]. In addition, it is suggested that younger people consider more about the price of the foods to be chosen, as they are likely to have other expenses that they deem to be a priority to the detriment of food.

From the observed results, it is also suggested that elderly people do not resort to the act of eating as a way to evade feelings, probably due to the greater risk of diseases and associated complications as age increases, which leads to the belief that this practice can lead to the worsening of the hepatitis and impair the treatment.

Furthermore, an inverse correlation was observed between education and the convenience scale, evidencing that the lower the education of the individuals, the greater the relevance of the more practical aspects of food for this group. Convenience presupposes the choice of food due to the speed of access or preparation, requiring minimal effort [15]. This scale reflects the make-up of modern food, considering the ingredients used, the preparation of the food, the place where the food was purchased and where it was consumed [22].

Therefore, people with a lower level of education may have multiple workdays or other tasks necessary for survival, and consequently need practicality in terms of food, which, according to Marsola et al. [23], can be considered a health-related risk behavior as it predisposes to poor diet quality from the consumption of ultra-processed foods. Garcêz et al. [24] cite in their study that the consumption of ultra-processed foods may play an important role in the development of non-alcoholic fatty liver disease, especially in overweight and obese individuals. Therefore, such a situation could contribute to worsening the clinical picture of the individual with viral hepatitis.

An inverse correlation was also observed between education and the social norms scale, which remained regardless of gender, income, education and origin. Social norms express the desire to present oneself favorably, being related to sociability [15] and to commensality that considers not only the food eaten, but also the eating habits involved with cultural and symbolic manifestations and rules of social organization [25]. 

Thus, it is suggested that the evaluated individuals are not influenced by the social context of food, probably because they know the impacts of food on the health/disease state, as well as because they probably have food taboos related to liver disease that make them afraid to consume supposedly “not allowed” foods, exacerbating the food dichotomy. 

An inverse correlation was observed between income and the food price scale, as observed in the study by Pechey et al. [17]. According to the 2017–2018 Household Budget Survey [26], families with lower incomes spend their income on purchasing food to survive, while families with higher income levels also carry out this purchase/consumption, but buy more expensive products. Therefore, it was shown that people with lower incomes are more concerned with the price of food when making food choices, when compared to people with higher incomes.

It is important to highlight that the present study was carried out during the period of the COVID-19 pandemic, which, according to the PENSSAN Network [27], was a period in which financial difficulties were intensified, favoring an increase in food insecurity rates.

Moreover, a positive correlation was observed between the individuals’ income and the weight control scale, similar to what was observed by Pechey et al. [17], indicating that economic status is an important factor for choosing foods based on weight control for people with viral hepatitis. Thus, it is suggested that individuals with higher incomes are able to have more access to information about health, which makes it possible to make decisions favorable to weight control and disease prevention.

Therefore, it is understood that excess body fat predisposes to health damage that culminates in impaired quality of life [28], in addition to bringing complications to viral hepatitis [29]. This suggests that income is a relevant aspect related to health literacy, which concerns skills related to access, understanding and use of information for health promotion [30].

It is important to highlight that the present study has the limitations of being a cross-sectional study with non-probabilistic convenience sampling. In addition, due to the data collection being carried out during the period of the COVID-19 pandemic, it is possible that there was a negative influence on the data collected. However, it is important to highlight that no studies were found that applied the TEMS to identify the reasons for food choices of people with viral hepatitis, emphasizing the importance of new studies that quantitatively assess not only food consumption, but also the determinants of food choices for these individuals, in order to develop practicable nutritional guidelines. In addition, new longitudinal studies are suggested to compare the motivations for food choices at the beginning and after treatment for viral hepatitis, with the objective of verifying whether the treatment provoked changes in the individual’s relationship with food.

## 5. Conclusions

From the weak correlations between the determinants of food choices and the socioeconomic and demographic factors of individuals with hepatitis B and C, it was observed that women and the elderly place more importance on the preference scale when choosing food. In addition, it was found that younger individuals consider emotional control as a determinant, using food as a mechanism to escape feelings, and they place more importance on the price of food.

Education was correlated with social norms and convenience, demonstrating that, as individuals attain higher education, less importance is given to the social context of food. On the other hand, individuals with less education are more attracted to convenience, preferring more practical foods.

The income of the research participants proved to be an influencing factor on the diet based on the weight control of these individuals, verifying that people with more favorable economic conditions have an eating pattern that considers the impact on body weight. Still, food choices based on food prices is an aspect influenced by the income of individuals, verifying that people with lower incomes consider food prices more.

Therefore, when learning about the determinants of food choices of people with hepatitis B and C and correlating these findings with the socioeconomic and demographic context in which individuals find themselves, it contributes to the elaboration of more realistic and feasible strategies, contributing to the autonomy of the individual in relation to their diet and favoring nutritional adherence as well as, consequently, effective changes in eating behavior.

## Figures and Tables

**Table 1:** Socioeconomic and demographic characterization and clinical characterization of individuals with viral hepatitis treated at a Reference Center in the Amazon, 2021.

	Mean/n	SD/%
Age	53.54	12.14
Gender		
Female	77	53.10
Male	68	46.90
Age range (years)		
23 to 44	35	24.14
45 to 59	55	37.93
60 to 74	55	37.93
Origin		
Capital or metropolitan region	114	78.62
Another municipality in Pará	30	20.69
Outside the State of Pará	1	0.69
Education		
Without higher education	119	82.07
With higher education	26	17.93
Income (MW)		
<1	85	58.62
2 to 3	47	32.41
4 to 5	6	4.41
>5	7	4.83
Diagnosis		
HBV	40	27.59
HCV	103	71.03
HBV + HCV	2	1.38

MW—minimum wage; HBV—hepatitis B virus; HCV—hepatitis C virus.

**Table 2:** TEMS dimension scores of individuals with viral hepatitis treated at a Reference Center in the Amazon, 2021.

	Mean	Min–Max	SD
Preference	12.11	6–15	2.60
Habits	12.43	5–15	2.25
Need and hunger	11.19	5–15	2.42
Health	10.66	3–15	2.60
Convenience	7.92	3–15	3.28
Pleasure	7.03	3–15	2.90
Traditional food	7.48	3–15	3.25
Natural issues	7.94	3–15	3.20
Socialization	5.99	3–15	2.72
Price	6.45	3–15	3.44
Visual attraction	4.23	3–15	2.20
Weight control	8.19	3–15	3.24
Emotion control	4.19	3–15	2.27
Social norms	6.23	3–15	2.42
Social image	3.60	3–10	1.23

**Table 3:** Correlation between socioeconomic and demographic factors and TEMS scales of individuals with viral hepatitis treated at a Reference Center in the Amazon, 2021.

	r^2^	*p*-Value *
Gender		
Preference	0.193	0.020
Age		
Preference	0.177	0.033
Price	−0.204	0.014
Emotional control	−0.168	0.044
Education		
Convenience	−0.172	0.039
Social norms	−0.206	0.013
Income		
Price	−0.208	0.012
Weight control	0.186	0.025

* Spearman correlation.

**Table 4:** Correlation between the “price” scale and the age of individuals with viral hepatitis treated at a Reference Center in the Amazon, 2021.

	*β*	R^2^	CI 95%	*p*-Value *
			(Min.; Max.)	
Model 1				
Age	−0.199	0.033	−0.102; −0.010	0.017
Model 2				
Age	−0.190	0.029	−0.100; −0.007	0.023
Gender	−0.059		−1.529; 0.725	0.482
Model 3				
Age	−0.180	0.064	−0.097; −0.005	0.029
Gender	−0.015		−1.231; 1.032	0.862
Income	−0.207		−1.614; −0.191	0.013
Model 4				
Age	−0.188	0.061	−0.099; −0.007	0.024
Gender	−0.023		−1.300; 0.988	0.787
Income	−0.167		−1.588; 1.135	0.098
Education	−0.070		−2.359; 1.099	0.473
Model 5				
Age	−0.189	0.054	−0.102; −0.005	0.031
Gender	−0.023		−1.304; 0.993	0.789
Income	−0.167		−1.594; 0.139	0.099
Education	−0.070		−2.364; 1.107	0.475
Origin	−0.004		−1.373; 1.316	0.967

* Linear regression model; dependent variable: “price” scale in The Eating Motivation Survey; co-variables: age, gender, income, education and origin.

**Table 5:** Correlation between the “emotional control” scale and the age of individuals with viral hepatitis treated at a Reference Center in the Amazon, 2021.

	*β*	R^2^	CI 95%	*p*-Value *
			(Min.; Max.)	
Model 1				
Age	−0.234	0.048	−0.074; −0.014	0.005
Model 2				
Age	−0.220	0.052	−0.071; −0.011	0.008
Gender	−0.100		−1.188; 0.283	0.226
Model 3				
Age	−0.217	0.049	−0.071; −0.010	0.009
Gender	−0.086		−1.144; 0.363	0.307
Income	−0.064		−0.659; 0.288	0.441
Model 4				
Age	−0.215	0.042	−0.071; −0.009	0.011
Gender	−0.085		−1.146; 0.380	0.323
Income	−0.073		−0.784; 0.365	0.473
Education	0.014		−1.068; 1.238	0.884
Model 5				
Age	−0.207	0.036	−0.071; −0.006	0.020
Gender	−0.085		−1.152; 0.379	0.320
Income	−0.071		−0.781; 0.373	0.486
Education	0.014		−1.075; 1.239	0.889
Origin	0.026		−0.760; 1.032	0.764

* Linear regression model; dependent variable: “emotional control” scale in The Eating Motivation Survey; co-variables: age, gender, income, education and origin.

**Table 6:** Correlation between the “social norms” scale and the education of individuals with viral hepatitis treated at a Reference Center in the Amazon, 2021.

	*β*	R^2^	CI 95%	*p*-Value *
			(Min.; Max.)	
Model 1				
Education	−0.201	0.034	−2.278; −0.245	0.015
Model 2				
Education	−0.201	0.027	−2.282; −0.241	0.016
Gender	−0.004		−0.804; 0.765	0.960
Model 3				
Education	−0.216	0.056	−2.367; −0.350	0.009
Gender	0.024		−0.665; 0.897	0.769
Age	−0.192		−0.070; −0.006	0.021
Model 4				
Education	−0.215	0.050	−2.574; −0.128	0.031
Gender	0.025		−0.691; 0.928	0.773
Age	−0.191		−0.071; −0.005	0.023
Income	−0.002		−0.616; 0.603	0.982
Model 5				
Education	−0.216	0.046	−2.585; −0.134	0.030
Gender	0.023		−0.701; 0.921	0.789
Age	−0.173		−0.069; 0.000	0.050
Income	0.002		−0.606; 0.617	0.985
Origin	0.060		−0.615; 1.284	0.487

* Linear regression model; dependent variable: “social norms” scale in The Eating Motivation Survey; co-variables: education, gender, age, income, and origin.

## Data Availability

The data presented in this study are available on request from the corresponding author.
